# The Molecular and Functional Characterization of the Durum Wheat Lipoxygenase TdLOX2 Suggests Its Role in Hyperosmotic Stress Response

**DOI:** 10.3390/plants9091233

**Published:** 2020-09-18

**Authors:** Valeria Menga, Daniela Trono

**Affiliations:** Consiglio per la Ricerca in Agricoltura e l’Analisi dell’Economia Agraria, Centro di Ricerca Cerealicoltura e Colture Industriali, S.S. 673, Km 25,200, 71122 Foggia, Italy; valeria.menga@crea.gov.it

**Keywords:** durum wheat, lipoxygenase, malondialdehyde content, osmotic stress, salt stress, superoxide anion production

## Abstract

In plants, lipoxygenases (LOXs) are involved in various processes, such as growth, development, and response to stress cues. In the present study, the expression pattern of six durum wheat LOX-encoding genes (*TdLpx-B1.1*, *TdLpx-B1.2*, *TdLpx-A2*, *TdLpx-B2*, *TdLpx-A3* and *TdLpx-B3*) under hyperosmotic stress was investigated. With osmotic (0.42 M mannitol) and salt (0.21 M NaCl) stress imposed at the early stages of seedling growth, a strong induction of the *TdLpx-A2* gene expression in the shoots paralleled an equally strong increase in the LOX activity. Enhanced levels of malondialdehyde (MDA) and increased rates of superoxide anion generation were also observed as a result of the stress imposition. Sequence analysis of the TdLOX2 encoded by the *TdLpx-A2* gene revealed that it belonged to the type-1 9-LOX group. When overexpressed in *E. coli*, TdLOX2 exhibited normal enzyme activity, high sensitivity to specific LOX inhibitors, with 76% and 99% inhibition by salicylhydroxamic and propyl gallate, respectively, and a preference for linoleic acid as substrate, which was converted exclusively to its corresponding 13-hydroperoxide. This unexpected positional specificity could be related to the unusual TV/K motif that in TdLOX2 replaces the canonical TV/R motif of 9-LOXs. Treatment of seedlings with propyl gallate strongly suppressed the increase in LOX activity induced by the hyperosmotic stress; the MDA accumulation was also reduced but less markedly, whereas the rate of superoxide anion generation was even more increased. Overall, our findings suggest that the up-regulation of the *TdLpx-A2* gene is a component of the durum wheat response to hyperosmotic stress and that TdLOX2 may act by counteracting the excessive generation of harmful reactive oxygen species responsible for the oxidative damages that occur in plants under stress.

## 1. Introduction

As sessile organisms, plants face various unfavourable environmental conditions. Salinity of soil is one of the most serious problems that limit agriculture in arid and semiarid areas [1]. Salt stress induces osmotic stress and ion toxicity inside the plant cells, which alter plant physiological and metabolic processes. These primary effects can in turn have secondary consequences, such as the accumulation of excessive reactive oxygen species (ROS). ROS regulate different physiological processes in plants; however, their overproduction may cause a variety of harmful effects on plant cell metabolism, thereby restricting plant growth and development, and reducing yield [2,3,4]. Therefore, plants have evolved different mechanisms to minimize the accumulation of ROS in the cells [5].

Lipoxygenase (LOX) (linoleate:oxygen oxidoreductase; EC 1.13.11.12) is a non-heme iron-containing dioxygenase that catalyze the incorporation of molecular oxygen into polyunsaturated fatty acids that contain one or more 1,4-*cis*,*cis* pentadiene moieties [6]. Depending on the carbon chain position at which oxygen is added, plant LOXs are distinguished into 9-LOXs and 13-LOXs, whose primary product is the 9- and 13-hydroperoxy fatty acid, respectively; LOX enzymes also exist that can produce both 9- and 13-hydroperoxides [7]. Moreover, on the basis of their primary structure and sequence similarity, these enzymes are also distinguished as type 1 and type 2 LOXs. The type 1 LOXs, which include both 9- and 13-LOXs, are highly similar to each other (>75%) and do not possess a transit peptide; conversely, the type 2 LOXs consist exclusively of 13-LOXs, are moderately similar to each other (35%) and present the N-terminal transit peptide for chloroplast targeting [7].

In plants, LOX enzymes are encoded by multi-gene families and the hydroperoxy fatty acids produced by LOXs are rapidly converted into diverse oxylipins [8], which have been shown to have roles in several biological events of plants. Accumulating evidence indicates that LOXs play a role in plant growth [9,10,11,12], arbuscular mycorrhiza formation [13], wounding [14,15] and defence to pathogens [16,17]. Moreover, numerous studies have found that LOXs are involved in plant response to abiotic stresses and, in particular, to hyperosmotic stresses. For example, an up-regulation of the *PgLOX3* gene was observed in the adventitious roots of ginseng plants exposed to water deficit [18], whereas pepper *CaLOX1* was found to be induced in leaves of plants grown under salt and drought stress [19]; *Arabidopsis* plants that overexpressed the *CaLOX1* gene were found to be more tolerant to ABA, mannitol and salt stress compared to wild-type plants [19]. An enhanced tolerance to osmotic stress, high salinity and drought was also observed in *Arabidopsis* plants that overexpressed the *DkLOX3* from persimmon [20]. Sixty four putative *GhLOX* genes were identified in four cotton species and some of these genes were found to be up-regulated in response to salt stress; the silencing of the *GhLOX12* and *GhLOX13* genes induced and increased susceptibility of cotton plants to salinity [21]. In the oriental melon, the promoter of *CmLOX08* gene was cloned, and *cis*-regulatory elements were identified that responded to different abiotic stresses including salt and drought [22].

Evidence also exists that salinity may induce significant increases in LOX activity. Exposure of soybean seedlings to mannitol stress was found to induce an increase of about 60% in the enzymatic activity and the protein levels of both LOX1 and LOX2 [23]. Significant increases in LOX activity were observed in leaves and roots of olive trees [24], as well as in tomato calli [25] exposed to progressively increasing water stress conditions. An increase in LOX activity was also detected in rice seedlings [26] and tomato plants [27,28] exposed to salinity.

Durum wheat (*Triticum durum* Desf.) is a typical crop widely grown in the Mediterranean region, where salt stress is caused by both the groundwater salinization in aquifers and the use of brackish water for irrigation [29]. Being a winter crop, durum wheat faces the highest salinity levels at seedling stage, when the soil contains high salt concentrations due to the low rainfalls and the high evaporation in the previous summer that induces the migration of salts to the soil surface [30]. Generally, durum wheat is considered to be moderately tolerant to salinity, but its productivity and quality can be significantly compromised by salt stress [31]. For this reason, the understanding of the mechanisms underlying durum wheat seedling responses to salinity represents an important goal to be pursued.

Among cereals, LOX genes and isoforms have been deeply investigated in barley. This crop possesses three LOX genes, namely *LoxA*, *LoxB* and *LoxC*, [32,33]. The *LoxA* and *LoxB* genes map to the 4HS chromosome, whereas the *LoxC* gene maps to the 7HL chromosome [33]. Two isoforms, LOX-1 and LOX-2, encoded by the *LoxA* and *LoxC* genes, respectively, have been purified and characterized [34]; conversely, the LOX isoform encoded by the *LoxB* gene has not yet been isolated. In durum wheat, LOX-encoding (*Lpx*) genes corresponding to the barley *Lox* genes have been identified. Three *Lpx-1* genes, namely *Lpx-B1.1*, *Lpx-B1.2* and *Lpx-B1.3*, orthologous to the barley *LoxA* gene, have been mapped to the 4B chromosome [35,36], a *Lpx-1_like* pseudogene has been identified on chromosome 4A [37], two partial sequences designated *Lpx-A3* and *Lpx-B3* with high similarity with barley *LoxB* have been assigned to the group 4 chromosomes, and two partial sequences designed *Lpx-A2* and *Lpx-B2* and corresponding to the barley *LoxC* gene have been mapped to the group 5 chromosomes [35].

To date, the studies on the durum wheat *Lpx* genes (hereafter referred to as *TdLpx* genes) have been focused exclusively on their involvement in the oxidative degradation of carotenoids that occurs during pasta processing and that leads to the bleaching of dough and pasta products [35,36,38]. By contrast, no information is still available about the role that LOXs could play in the durum wheat response to environmental stresses. So, to shed some light into this aspect, in the present study an investigation was carried out on durum wheat seedlings to evaluate changes in the *TdLpx* genes expression and the LOX activity in response to mannitol and salt stress and in relation to ROS accumulation and membrane lipid peroxidation. To do this, the durum wheat cv. ‘Ofanto’ was chosen on the basis of its tolerance to salinity previously assessed by our research group. Among different genotypes exposed to different salinity levels, the cv. ‘Ofanto’ showed the highest retention of total biomass [39] and the maintenance of good grain yields [40]; these observations suggest the ability of this genotype to cope with salt stress by activating mechanisms at cellular level able to limit the detrimental effects of salinity on plant growth. The results reported in the present paper provide evidence for a role of the *TdLpx-A2* gene in the durum wheat response to hyperosmotic stress and suggest that the TdLOX2 isoform might act by controlling the ROS accumulation that under stress is responsible for the increased levels of non-enzymatic peroxidation of membrane lipids.

## 2. Results and Discussion

### 2.1. Growth Performances of Seedlings Grown under Control, Mannitol and NaCl Stress Conditions

**Figure 1:**
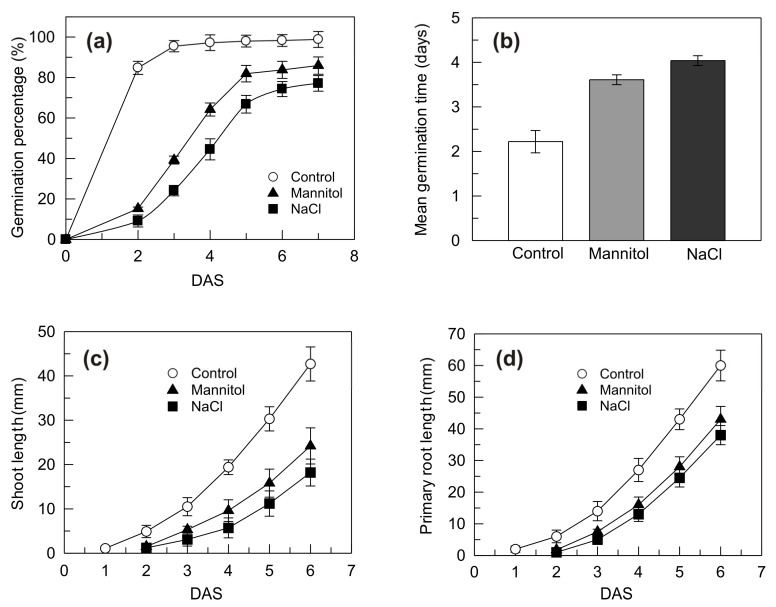
Germination percentage (**a**), mean germination time (**b**), shoot length (**c**) and primary root length (**d**) of durum wheat seedlings grown under control, mannitol and NaCl stress conditions. DAS, days after sowing. Vertical bars represent ± S.D. (n = 3 independent experiments).

An experiment was carried out to evaluate the damages induced to seedling germination and growth by mannitol and NaCl stress (Figure 1). Under the control condition, the percentage of germination rapidly increased in the very first days after sowing (DAS), reaching 80% at the 2nd DAS and more than 98% at the end of the period examined (Figure 1a). Compared to the control condition, both osmotic and salt stress significantly delayed germination and reduced the germination percentage that was only 15% and 9%, respectively, at the 2nd DAS and reached 84% and 74%, respectively, at the end of the period examined (Figure 1a). Consistently, under the stress conditions, a significant increase was observed on the mean germination time from 2.22 days under the control condition to 3.61 days and 4.04 days under osmotic and salt stress, respectively (Figure 1b). In both cases the adverse effects were slightly greater in NaCl than in mannitol-treated seedlings. Mannitol and NaCl stress negatively affected also the seedling growth, although the effects of the two treatments did not differ significantly between each other (Figure 1c,d). Compared to the control condition, the shoot length was roughly halved under both stresses (Figure 1c). The effect of the stress imposition on the length of the primary root was less evident, with a one-third reduction observed over the period examined (Figure 1d).

These results agree with previous observations, which showed that osmotic stress and salinity in the immediate environment of germinating durum wheat seeds can delay or inhibit the germination process [41,42] and reduce the early seedling growth [43,44,45]. The use of a mannitol solution iso-osmotic to the NaCl solution allows to evaluate the relative contribution of the two components of the salt stress, namely the osmotic stress and the ion toxicity. So, the observation that no great differences exist between damages due to mannitol and NaCl treatment suggests that the adverse effects on germination and early seedling growth are mainly due to the osmotic effect rather than to the ion-toxic effect of NaCl. This result agrees with the two-phase response of wheat growth to salinity reported by Munns [3] according to which, in wheat plants, osmotic stress inhibits growth in the early days of salt stress treatment while the toxic stress, due to salt accumulation inside the cells, exhibits its effects later.

### 2.2. Malondialdehyde Accumulation and Superoxide Anion Generation Rate in the Shoots of Seedlings Grown under Control, Mannitol and NaCl Stress Conditions

**Figure 2:**
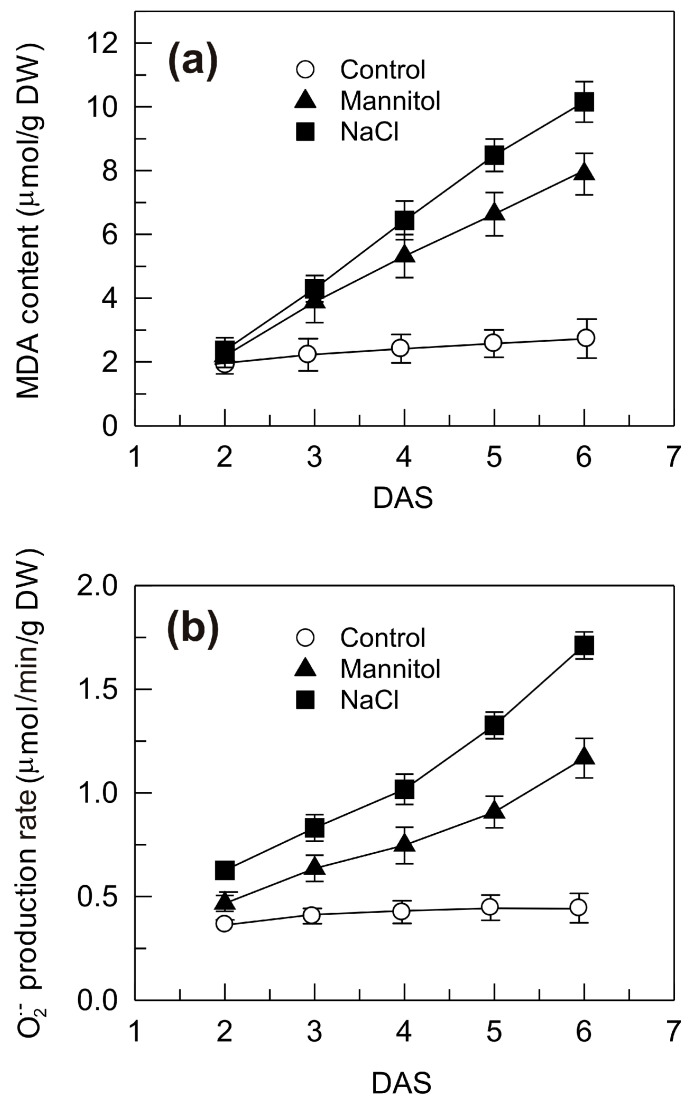
Malondialdehyde (MDA) accumulation (**a**) and superoxide anion (O_2_^•−^) production rate (**b**) in the shoots of durum wheat seedlings grown under control, mannitol and NaCl stress conditions. DAS, days after sowing. Vertical bars represent ± S.D. (n = 3 independent experiments).

It is widely ascertained that the osmotic stress and the ion-toxic effect induced by salinity in plant cells result in the oxidative stress, which can damage cellular components, including membrane lipids [46]. In this context, the content of malondialdehyde (MDA), which is a well-known lipid oxidation marker [47], and the rate of superoxide anion generation were measured in the shoots of seedlings grown under control conditions and exposed to osmotic and salt stress (Figure 2). Under the control condition, no significant variations in the MDA content were detected over the period examined, whereas a strong increase was observed under the hyperosmotic stresses (Figure 2a). At the end of the period examined, the MDA concentrations reached under mannitol and salt stress were 193% and 272% higher, respectively, than that in the control shoots (Figure 2a). Similarly, the rate of superoxide anion production under the control condition remained almost unchanged all over the period examined, whereas the hyperosmotic stresses caused a strong increase that became more evident as the time of exposure increased (Figure 2b). At the end of the period examined, the rate of superoxide anion production under mannitol and salt stress reached values 167% and 292% higher, respectively, than that in the control shoots (Figure 2b).

These results indicate that under normal growth conditions plants tend to maintain a balanced redox status. Hyperosmotic stresses may disturb this balance by inducing ROS accumulation and peroxidation of membrane lipids. These results are in line with previous studies that reported significant elevations of MDA and ROS in wheat genotypes exposed to drought or salinity [48]. Interestingly, both the MDA content and the superoxide anion production rate were inhibited more by NaCl than mannitol stress, which indicated that the redox status was negatively affected by both the osmotic and ion-toxic component of the salt stress.

### 2.3. Expression Levels of the TdLpx Genes in the Shoots of Seedlings Grown under Control, Mannitol and NaCl Stress Conditions

**Figure 3:**
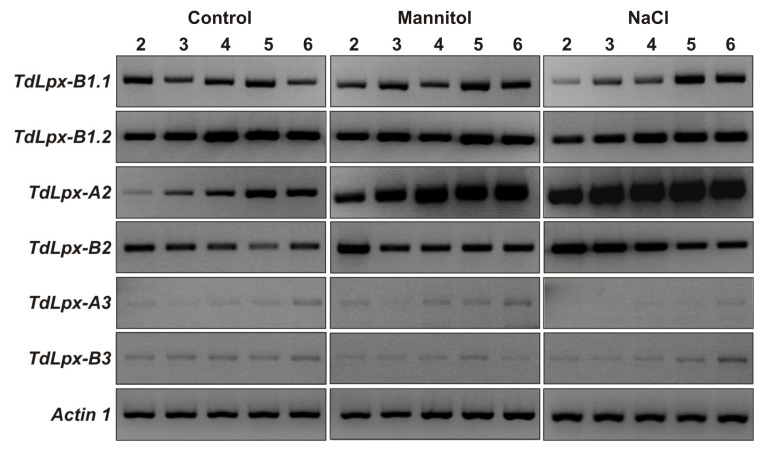
Expression analysis of the *TdLpx* genes under control, mannitol and NaCl stress conditions. Total RNA was isolated from shoots of durum wheat seedlings grown under control and stress conditions and subjected to semiquantitative RT-PCR analysis using the specific primer pairs and the amplification conditions reported in Methods. Normalization of the PCR reactions was performed by amplification of the constitutive *actin1* gene. The number on each lane represents the day after sowing at which shoots were harvested.

Evidence exists that LOXs are involved in the alteration of the redox status and in the modification of the membrane lipids in the cells of plants exposed to hyperosmotic stresses [23]. This prompted us to evaluate the expression profiles of the LOX-encoding genes, *TdLpx-1*, *TdLpx-2* and *TdLpx-3*, in the shoots of durum wheat seedlings exposed to mannitol and NaCl stress (Figure 3).

Under the control condition, all the *TdLpx-1 and TdLpx-2* genes were expressed at considerable levels, with the highest levels observed for the *TdLpx-B1.2* gene; very low or even undetectable levels were instead observed for the *TdLpx-3* genes (Figure 3). The transcript levels remained relatively constant all over the period examined for all the genes except the *TdLpx-A2* gene, whose expression increased over time (Figure 3). A similar expression pattern has been reported for the orthologous genes in barley, a cereal grain species highly similar to wheat. In this species, the transcripts corresponding to the *LoxA* and *LoxC* genes, orthologous to the durum wheat *TdLpx-1* and the *TdLpx-2* genes, respectively, were found to be significantly present in the shoots from day 2 until day 5 after germination, whereas the transcripts corresponding to the *LoxB* gene, orthologous to the *TdLpx-3* genes, were poorly detected [33,34].

Under both mannitol and NaCl stress, the expression levels of the *TdLpx-1* and *TdLpx-3* genes remained almost unchanged compared to the control condition, with the remarkable exception of a slight induction observed for the *TdLpx-B1.1* gene between the 5th and the 6th DAS (Figure 3). As regards the *TdLpx-2* genes, a slightly down-regulation was observed for the *TdLpx-B2* gene under salinity, whereas the *TdLpx-A2* was found to be strongly up-regulated under both mannitol and salt stress, with the transcript levels that increased with the time of exposure to the stress (Figure 3). Since the beginning of the treatment a stronger effect was observed under NaCl compared to mannitol stress.

The pattern revealed by the expression analysis clearly showed that, among all the *TdLpx* genes, the *TdLpx-A2* gene is responsible for playing a specific role in the durum wheat response to hyperosmotic stresses. This is consistent with the increasing observations that the LOX gene families in plants include genes that have different expression patterns under various endogenous and external stimuli, which may reflect their involvement in specific growth- and stress-related processes. For instance, the LOX gene families of ginseng [18], cotton [21] and poplar [49] have been deeply characterized and the expression analysis has revealed that some LOX genes are specifically regulated in response to phytohormones, pathogens, wounding, and abiotic stresses. In this context, the results reported in the present study extend these knowledges to a monocot species in which the induction of LOX-encoding genes under environmental cues has never been explored previously.

### 2.4. LOX Activity in the Shoots of Seedlings Grown under Control, Mannitol and NaCl Stress

**Figure 4:**
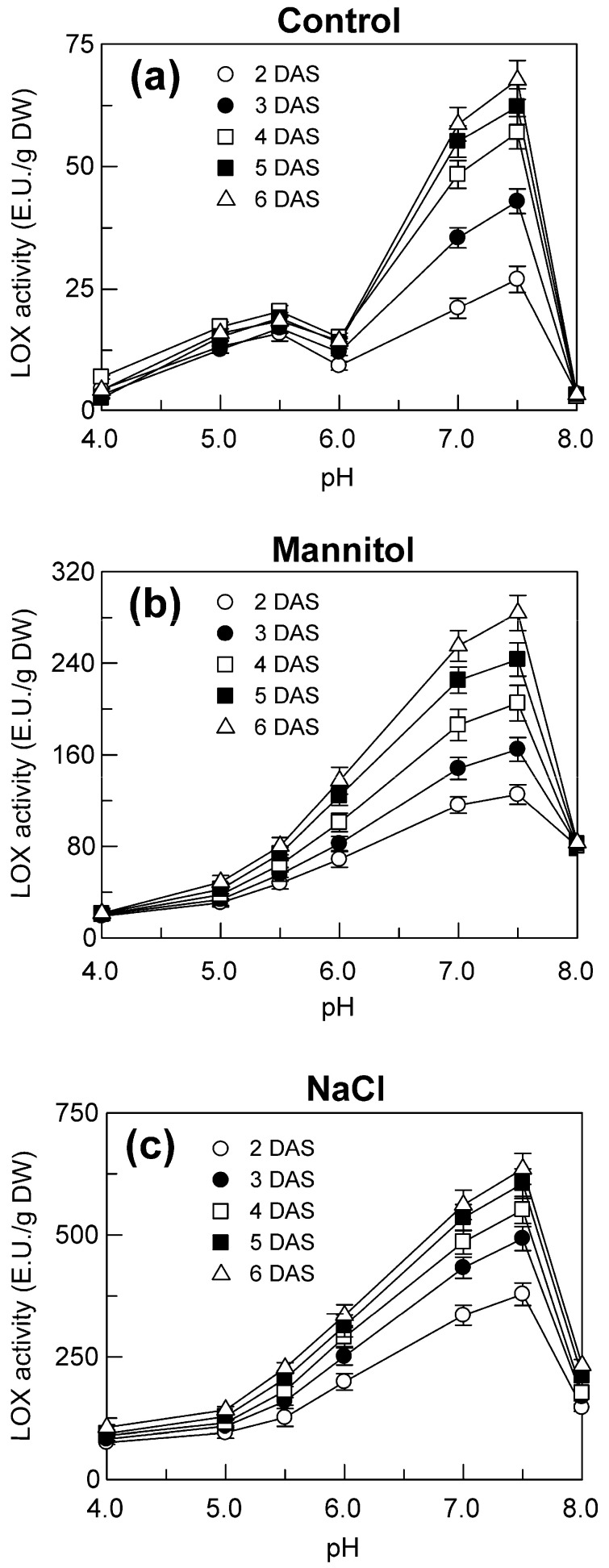
LOX activity in the crude extract from the shoots of durum wheat seedlings grown under control (**a**), mannitol (**b**) and NaCl stress (**c**) conditions. Measurements were carried out as described in Methods using 1 mM Na linoleate as substrate. The buffers were as follows: 50 mM Na acetate, pH 4.0–5.5; 50 mM Na phosphate, pH 6.0–8.0. Vertical bars represent ± S.D. (n = 3 independent experiments).

To check if the increase in the *TdLpx-A2* transcript levels observed under stress was accompanied by a concomitant increase in the enzymatic activity, an experiment was carried out to evaluate the LOX activity in the shoots of durum wheat seedlings grown under control and stress conditions (Figure 4).

Under the control condition, the LOX activity showed two peaks, the smaller at pH 5.5, and the higher at pH 7.5 (Figure 4a), which suggested the presence in this tissue of at least two different LOX isoforms. The peak at pH 5.5 is compatible with the optimum observed at pH 5.3 in durum wheat semolina [38] and it is probably ascribable to the LOX isoforms present also in mature grains. Our previous findings revealed that the *TdLpx-1* and *TdLpx-3* genes were the only *TdLpx* genes expressed in mature grains [36,50]. Since in the shoots the *TdLpx-3* genes are expressed at very low levels or are not expressed at all, it can be assumed that the LOX activity that peaked at pH 5.5 is due to the LOX1 isoforms encoded by *TdLpx-1* genes. Conversely, the peak at pH 7.5, which has been not detected in durum wheat mature grains [36,50], is probably due to the TdLOX2 isoforms whose encoding genes (*TdLpx-2*) are expressed in the shoots but not in the mature grains.

In this regard, evidence has been reported in barley that the LOX2 isoform presented a broad peak at pH 7.0 [51], and its activity and protein levels increased in the coleoptiles during the first days of germination [34]. Consistent with these previous findings and with the increase in the expression of the *TdLpx-A2* gene detected under the control condition (Figure 3), we observed an increase in the LOX activity at pH 7.5 that was particularly evident between the 2nd and the 4th DAS and continued at a slower rate until the end of the period examined, when the activity reached, on average, a value 48% higher than that observed at the 2nd DAS (Figure 4a). No significant variations were detected during seedling growth in the activity at pH 5.5 (Figure 4a), which is consistent with the stable transcript levels of the *TdLpx-1* genes detected under the control condition (Figure 3).

Under mannitol and NaCl stress, a strong increase in the LOX activity that peaked at pH 7.5 occurred; in both cases, the increase was so great that it led to the complete disappearance of the peak at pH 5.5, which most likely remained unchanged under stress (Figure 4b,c). As already observed for the up-regulation of the *TdLpx-A2* gene, NaCl had greater effect than mannitol. The increase observed under NaCl stress was particularly evident at the onset of germination (between the 2nd and the 3rd DAS), whereas mannitol stress caused a gradual increase over time (Figure 4b,c). At the end of the period examined, the LOX activity under mannitol stress was on average threefold higher than that measured under the control condition, whereas an average ten-fold increase was observed under salinity (Figure 4b,c).

These findings demonstrate that the seedling growth process, as well as the hyperosmotic stress imposition, cause variations in the enzyme activity that are comparable to those observed for the expression levels of the *TdLpx-A2* gene. Such a closely coupled relationship strengthen our hypothesis that the TdLOX2 isoform encoded by the *TdLpx-A2* gene is responsible for the LOX activity that peaks at pH 7.5. However, this straight relationship should be seen with caution. In addition to the increase in the *de novo* synthesis of the TdLOX2 isoform due to the induction of *TdLpx-A2* gene expression, a regulation of the enzyme at post-transcriptional and/or post-translational level cannot be excluded. At this regard, it is known that in plants exposed to stress conditions LOXs can be regulated by other mechanisms besides gene induction, which include the alternative splicing, phosphorylation, allosteric modulation and protein-protein interaction [52]. Anyway, our findings represent a relevant progress on this topic because they represent the first detailed evidence of the significance of LOXs in general, and of the TdLOX2 in particular, in the physiological processes and the stress-related responses of durum wheat. The only other observation on this species was reported by our research group and concerned the involvement of LOX activity in the process of leaf senescence [53]. Whether the TdLOX2 induction is a genotype-specific response that contributes to the salinity tolerance of the cv. ‘Ofanto’ remains to be established. Future studies on the comparison of different genotypes could help to clarify this point.

It is noteworthy that the *TdLpx-A2* gene expression and the LOX activity, similarly to the MDA content and the superoxide anion generation, are affected by both the osmotic and the ion-toxic component of the NaCl stress. This observation raises the possibility that these metabolic responses are somehow related to each other and indicates that the pathway in which they are possibly involved represents a sensitive target of the toxic stress.

### 2.5. Isolation and Characterization of the Full-Length TdLpx-A2 Transcript

To investigate about the role of the TdLOX2 isoform in the durum wheat response to hyperosmotic stresses, the full-length coding sequence of the *TdLpx-A2* gene was isolated after the cDNA amplification from the shoots of control durum wheat seedlings at the 2nd DAS. The sequence was deposited at NCBI GenBank database under the accession number MT843321. The full-length *TdLpx-A2* transcript harboured an open reading frame (ORF) of 2595 nucleotides encoding a putative translation product of 864 amino acids with a molecular mass of about 96.75 kDa and a pI of about 6.1, estimated using the ProtParam tool [54].

**Figure 5:**
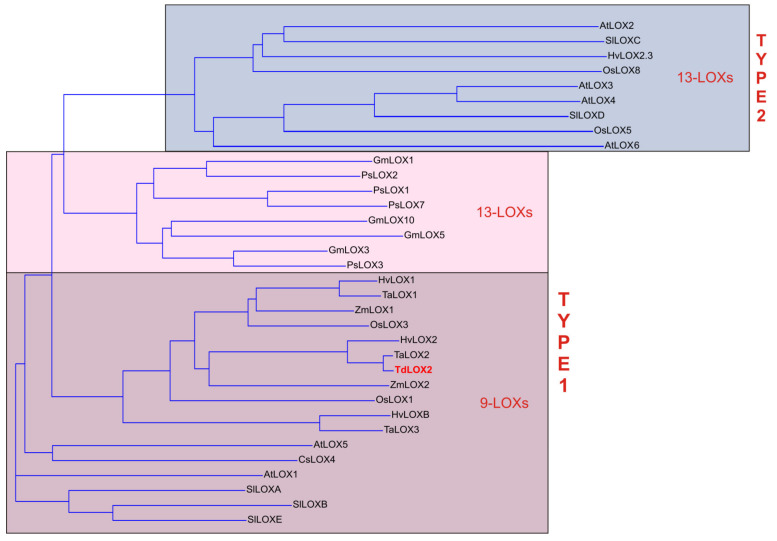
Phylogenetic tree analysis of the deduced amino acid sequences of selected plant LOXs. Alignments were calculated using Vector NTI Suite software (Ver. 10.3.0, Thermo Fisher Scientific, Waltham, MA, USA). The NCBI GenBank accession numbers of the LOXs included in the analysis are as follows: *Arabidopsis thaliana* (AtLOX1, Q06327; AtLOX2, P38418; AtLOX3, Q9SMW1; AtLOX4, Q9FNX8; AtLOX5, Q9FNX7; AtLOX6, Q9CAG3), *Cucumis sativus* (CsLOX4, CAB83038), *Glycine max* (GmLOX1, P08170; GmLOX3, P09186; GmLOX5, AAB67732; GmLOX10, ABS32276), *Hordeum vulgare* (HvLOX1, AAA64893; HvLOX2, AAB70865; HvLOX2.3, Q8GSM2; HvLOXB, AAB60715), *Oryza sativa* (OsLOX1, Q76I22; OsLOX3, Q7G794; OsLOX5, XP_015637182; OsLOX8, XP_015650450), *Pisum sativum* (PsLOX1, AB71759; PsLOX2, P14856; PsLOX3, P09918; PsLOX7, CAC04380), *Solanum tuberosum* (SlLOXA, P38415; SlLOXB, P38416; SlLOXC, Q96573; SlLOXD, Q96574; SlLOXE, Q43501), *Triticum aestivum* (TaLOX1, ACS34909; TaLOX2, ACS34908; TaLOX3, ADZ31265), *Triticum durum* (TdLOX2, MT843321), *Zea mais* (ZmLOX1, AAL73499; ZmLOX2, AAF76207).

The phylogenetic tree of the deduced amino acid sequence of the TdLOX2 identified in the present study, along with LOXs from other plant species, showed that the TdLOX2 belonged to the type-1 9-LOX group (Figure 5). As already observed, LOXs from monocots and dicots in this group formed two separated clusters [49,55], thus suggesting that these genes have evolved after monocot-dicot divergence. In the monocot cluster, TdLOX2 shared 60.5% to 98.6% identity with the other cereal LOXs, with the lowest identity shared with the barley LOXB (HvLOXB) and the highest with bread wheat LOX2 (TaLOX2). TdLOX2 shared also high identity (93.3%) with the barely LOX2 (HvLOX2).

**Figure 6:**
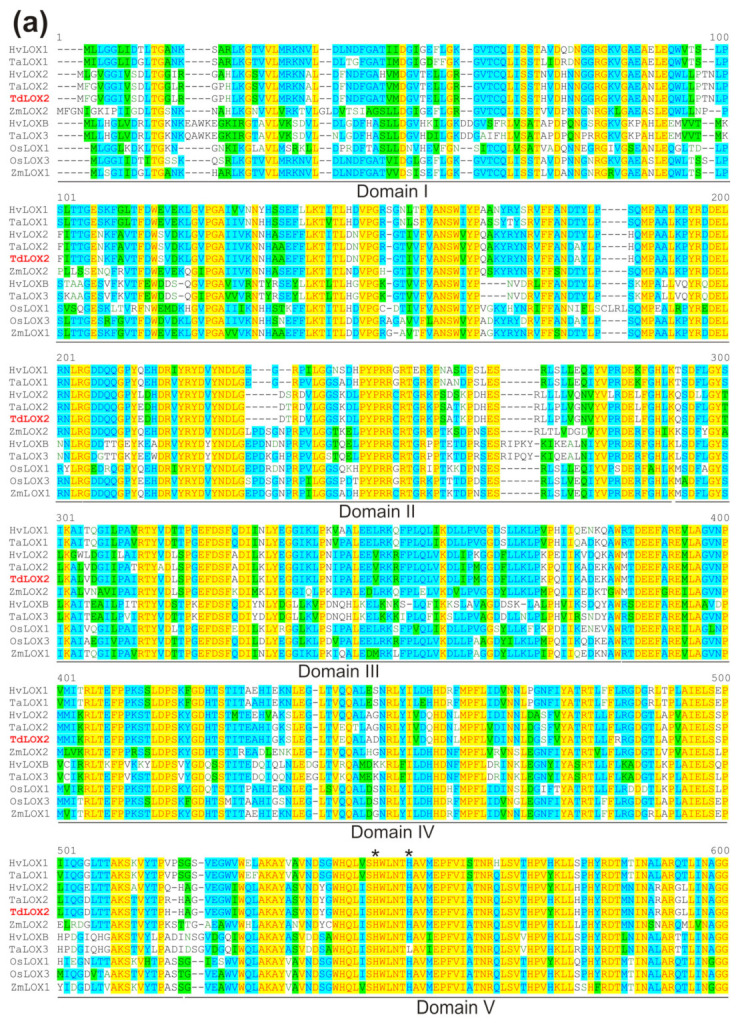

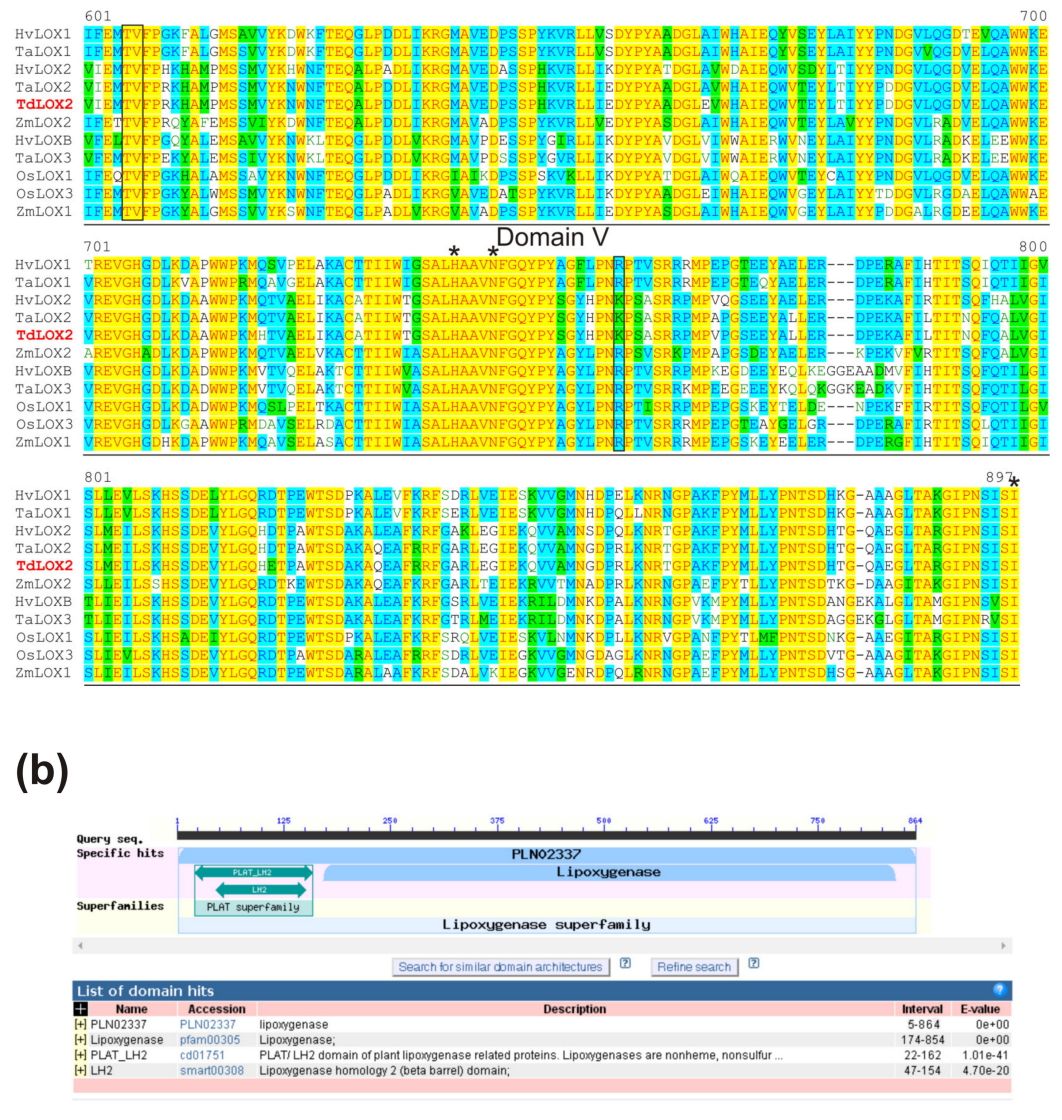
Analysis of the deduced amino acid sequence of the TdLOX2. Alignment of the deduced amino acid sequence of the TdLOX2 with other LOXs from monocots (**a**). Conserved domains (from I to V) as assigned by Minor and coworkers [56] are underlined. Asterisks indicate residues required for iron binding. The 9-LOX-specific TV/R motif is boxed. The alignments were performed using Vector NTI Suite software (Ver. 10.3.0, Thermo Fisher Scientific, Waltham, MA, USA). N-terminal PLAT domain and typical LOX domain in the TdLOX2 as identified by the NCBI Conserved Domain Search Database (http://www.ncbi.nlm.nih.gov/Structure/cdd/cdd.shtml) (**b**).

Highly conserved regions were identified in the TdLOX2 through comparison with 9-LOXs from other cereal species (Figure 6a,b): (i) the N-terminal PLAT/LH2 domain (domain I) that forms a β-barrel structure that targets the substrate to the active site; (ii) the C-terminal pfam00305 domain (domains II-V), which includes the conserved regions that are important for substrate and oxygen binding; and (iii) the hystidine (H520, H525 and H711), asparagine (N715), and isoleucine (I864) residues that are essential for iron binding. This organization is in line with the crystal structure determined by Minor and coworkers [56] for the soybean LOX1, which consisted of four smaller domains that associated on the surface of a larger C-terminal domain. Notably, TdLOX2, together with TaLOX2 and HvLOX2, differs from the other cereal 9-LOXs in that the arginine (R) residue in the TV/R motif, responsible for the positional specificity of 9-LOXs [7], is substituted by a lysine (K) residue (Figure 6a); this substitution gives rise to an unusual TV/K motif, which, to our knowledge, has not been described previously in other plant species.

A search for intracellular sorting and processing peptides (TargetP 1.1, http://www.cbs.dtu.dk/services/TargetP/; ipSORT, http://ipsort.hgc.jp/) in the predicted amino acid sequence of TdLOX2 suggested that this protein is probably located in the cytosol as it does not contain targeting or retention signals for any organelles. This is expected since the phylogenetic analysis revealed that TdLOX2 belonged to the type-1 LOX group.

### 2.6. Functional Characterization of the Recombinant TdLOX2

To assess the biochemical properties of the TdLOX2 encoded by the *TdLpx-A2* gene, the coding region of the gene was placed under the control of the L-(+)-arabinose promoter of an *E. coli* expression vector and the His-tagged protein was purified by Ni-NTA affinity chromatography. The recombinant protein was used to determine the substrate and product specificity of the TdLOX2, as well as its dependence on pH and its sensitivity to specific LOX inhibitors.

The Km and Vmax values obtained for linoleic, linolenic and arachidonic acids revealed that the TdLOX2 showed the highest substrate affinity and specific activity towards linoleic acid, although it also showed a good preference for linolenic acid; conversely, a very low affinity and specific activity towards arachidonic acid was observed (Table 1). These results indicated that the TdLOX2 preferred linoleic acid as substrate. The Km value for linoleic acid was of the same order of magnitude than that reported for the LOX2 isoform purified from germinated barley (74 µM vs. 180 µM) [57], although much lower values (16–19 µM) have been also reported for the barley LOX2 [51,58]. The observed differences may be ascribable to the different solubilization methods used for the preparation of the linoleate solution; so, comparison of the Km values obtained here and in other studies is not possible.

**Figure 7:**
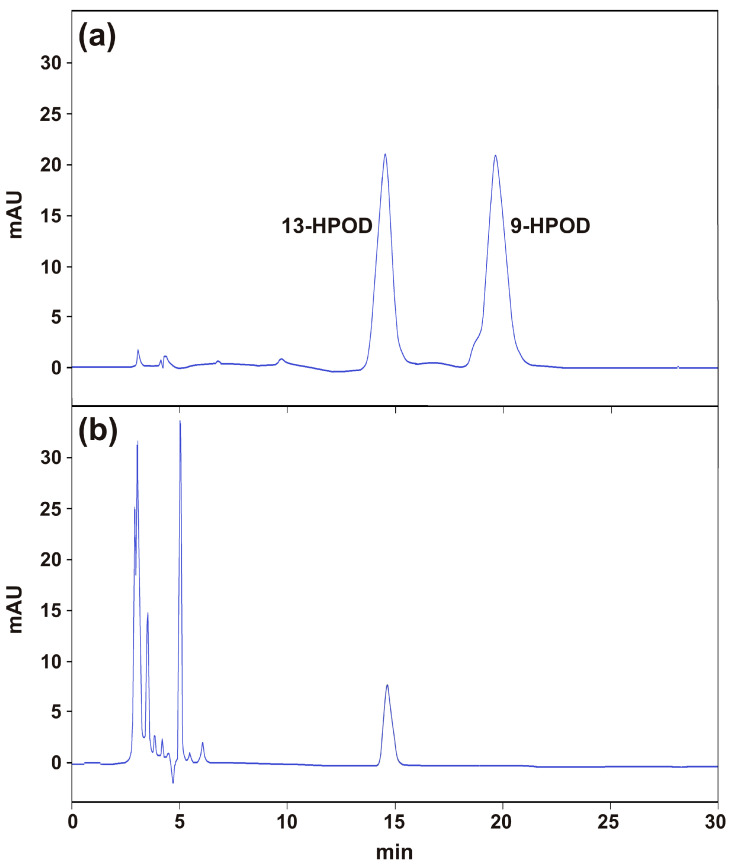
Determination of the positional specificity of the recombinant TdLOX2. Measurements were carried out as described in Methods. SP-HPLC analysis of a mixture of authentic 13- and 9-hydroperoxide derivatives of linoleic acid (13-HPOD and 9-HPOD) (**a**), and of the mixture of the reaction catalyzed by the recombinant TdLOX2 (**b**).

Given the preference of the TdLOX2 for linoleate, this was used as substrate for the evaluation of its product specificity, pH profile and sensitivity to specific LOX inhibitors. To study the product specificity, the reaction products of the TdLOX2 were separated by straight phase (SP)-HPLC. Surprisingly, according to the retention times of authentic 9- and 13-hydroperoxide derivatives of linoleic acid (HPOD), the recombinant TdLOX2 was found to produce exclusively 13-HPOD (Figure 7). This observation did not fit the positional specificity predicted by the phylogenetic analysis, which assigned the TdLOX2 to the type-19-LOX group. We can speculate that the presence in the TdLOX2 amino acid sequence of the unusual TV/K motif in place of the canonical TV/R motif could be responsible for this unexpected position specificity. This hypothesis is corroborated by previous findings that revealed the 13-HPOD as the only reaction product of the barley HvLOX2 [51], which shared with TdLOX2 and TaLOX2 the same unusual TV/K motif (Figure 6a). The LOX-derived 13-HPOD is the first substrate in the synthesis of jasmonic acid (JA) via allene oxide synthase and grean leaf volatiles (GLV) via hydroperoxide lyase. Unlike dicots, in which these two pathways are commonly co-localized to the chloroplast, in monocots evidence has been reported that only the JA biosynthesis takes place in the chloroplast, whereas the biosynthesis of GLV occurs outside the chloroplasts [59]. In the light of this, it might be possible that under stress TdLOX2 takes part to the GLV production. In this context, recent findings have demonstrated the protective and priming effect of GLV not only against pathogens and wounding but also against abiotic stresses [60] including salinity [61].

**Figure 8:**
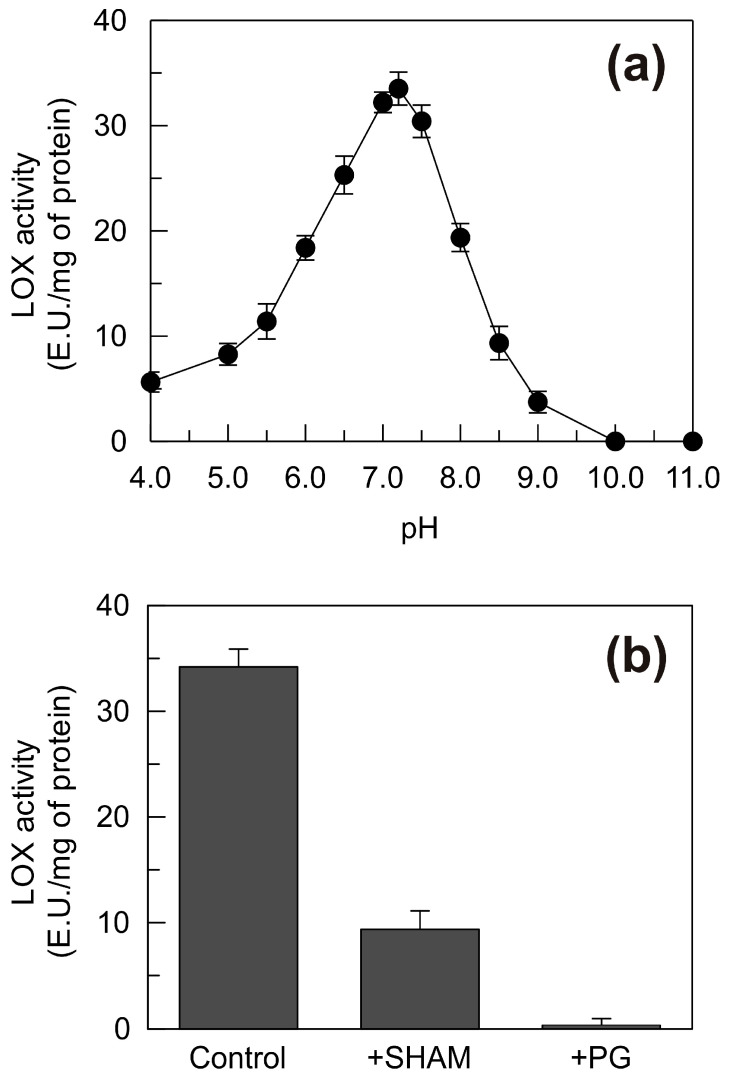
pH dependence (**a**) and sensitivity to inhibitors (**b**) of the TdLOX2 activity. Measurements were carried out as described in Methods using 1 mM Na linoleate as substrate. In (**a**) the buffers were as follows: 50 mM Na acetate, pH 4.0–5.5; 50 mM Na phosphate, pH 6.0–8.0; 50 mM Na borate, pH 9.0–11.0. In (**b**) the assays were carried out at pH 7.2; SHAM, salicylhydroxamic acid; PG, propyl gallate. Vertical bars represent ± S.D. (n = 3 independent experiments).

For the pH dependence, a single bell-shaped curve was observed (Figure 8a). The LOX activity of the recombinant protein increased in the pH range of 4.0 to 7.2, at which the activity reached its optimum; at higher pH values, the activity decreased to almost undetectable levels below pH 9.0 (Figure 8a). Interestingly, the pH range of action and the pH optimum of the recombinant TdLOX2 are comparable to those of the second higher peak detected for the LOX activity in the shoots (Figure 4). This further supports our belief that the up-regulation of the *TdLpx-A2* gene and the consequent *de novo* synthesis of the TdLOX2 enzyme represents the molecular mechanism responsible for the increase in the enzyme activity detected in the 6.0–8.0 pH range in the course of seedling growth and in response to hyperosmotic stress imposition (Figure 4).

The recombinant enzyme was found to be sensitive to the specific LOX inhibitors salicylhydroxamic acid (SHAM) and propyl gallate (PG) [62]. In particular, about 70% inhibition of the LOX activity was observed in the presence of 1 mM SHAM, whereas an almost complete inhibition (99%) occurred in the presence of 1 mM PG (Figure 8b).

### 2.7. Effect of PG on the LOX Activity, the MDA Content and the Rate of Superoxide Anion Generation in the Shoots of Seedlings Grown under Control, Mannitol and NaCl Stress Conditions

**Figure 9:**
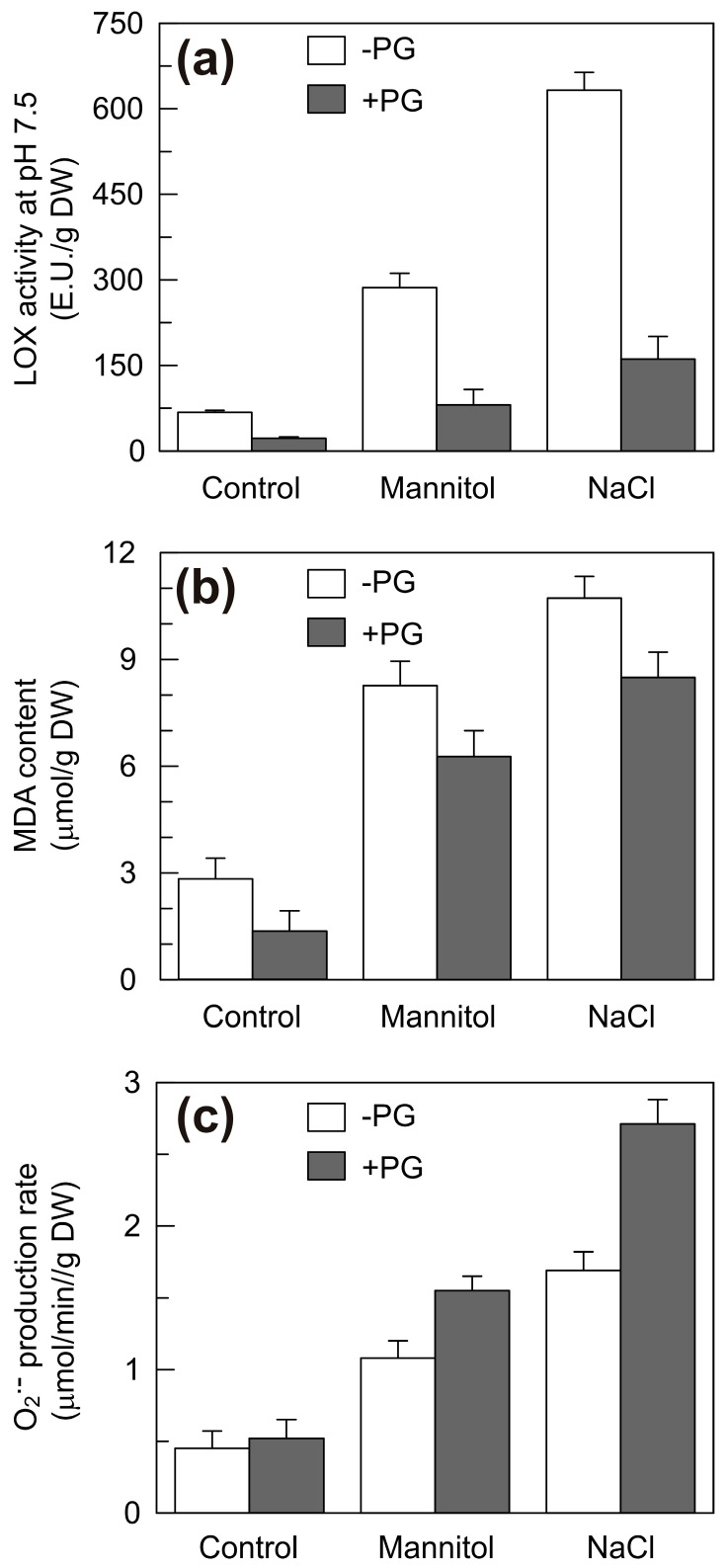
Effect of 1 mM propyl gallate (PG) on the LOX activity at pH 7.5 (**a**), the malondialdehyde (MDA) content (**b**) and the rate of superoxide anion (O_2_^•−^) production (**c**) in the shoots of durum wheat seedlings grown under control, mannitol and stress conditions at the 6th DAS. Vertical bars represent ± S.D. (n = 3 independent experiments).

To examine if and how inhibition of LOX activity in the shoots alters the accumulation of ROS and the peroxidation of membrane lipids promoted by hyperosmotic stresses, an experiment was carried out in which the seedling growth solutions (water, mannitol or NaCl) were supplemented with 1 mM PG. To monitor the activity ascribable to the TdLOX2, the enzymatic assays were carried out at pH 7.5. After 6 DAS, treatment of durum wheat seedlings with PG was accompanied by a strong inhibition of the LOX activity on average by 68%, 72% and 78% under control, mannitol and NaCl stress, respectively (Figure 9a). The MDA content was also significantly reduced by the PG treatment but to a lesser extent; in particular, an average 48% decrease was observed under the control condition that dropped to 21–24% under the stress conditions (Figure 9b).

Evidence exists that the LOX-catalyzed peroxidation of membrane lipids is a key mechanism in the adaptative response to abiotic and biotic stresses in different plants species [63]. Therefore, based on these observations, it was expected that the stress-induced MDA accumulation would be more strongly inhibited by PG treatment. Instead, our results show that the decrease in the membrane lipid peroxidation induced by PG under stress is limited compared to the decrease in the LOX activity observed under the same conditions. 

This suggests that TdLOX2 gives a minor contribution to the total membrane lipid peroxidation under hyperosmotic stresses, and raises the possibility that other mechanisms, such as the non-enzymatic oxidation induced by ROS accumulation, are involved.

As far as the rate of superoxide anion generation, the PG treatment caused no significant variation in the shoots of seedlings grown under the control condition but, surprisingly, a significant variation was observed under stress, with an average increase of 44% and 60% under mannitol and NaCl stress, respectively (Figure 9c). The increased rate of the superoxide anion generation observed under stress as a consequence of the LOX inhibition by PG suggests an involvement of the LOX activity in the control of the ROS generation induced by stress. The existence of a link between plant LOXs and ROS has been clearly demonstrated in transgenic plants subjected to hyperosmotic stresses. The increased tolerance to water deficit and high salinity shown by *Arabidopsis* plants overexpressing the pepper *CaLOX1* gene [19] or the persimmon *DkLOX3* gene [20] was found to be accompanied by a lower accumulation of ROS compared to the wild-type plants. In addition, the silencing of cotton *GhLOX12* and *GhLOX13*, as well as the overexpression of *ZmLOX1* gene in rice demonstrated that the involvement of LOXs in the control of ROS accumulation under hyperosmotic stresses was linked to the activation of the antioxidant enzymes superoxide dismutase, catalase and peroxidase [21,64].

Altogether, our findings suggest that under osmotic and salt stress ROS play a relevant role in the peroxidation of membrane lipids. The up-regulation of the *TdLpx-A2* gene and the concomitant increase in the TdLOX2 may represent a protective mechanism that keeps the stress-triggered ROS generation under control and prevent excessive accumulation of MDA. At low level, both MDA and ROS can have beneficial effects through the activation of regulatory genes involved in plant defense response and granting cell protection under oxidative stress conditions, which ultimately results in plant survival under stress [65]. Consistently, our results show that in durum wheat the *TdLpx-A2* expression and the TdLOX2 activity represent more sensitive targets of the salt stress-induced toxic effect than either germination or growth; so, it is feasible that their prompt activation under salinity might help to prevent ion-toxic inhibition of early seedling growth.

## 3. Materials and Methods

### 3.1. Growth of Control and Stressed Seedlings

Control seedlings were obtained by sowing durum wheat seeds from the cv. ‘Ofanto’ on Whatman filter paper supported by polyurethane foam saturated with distilled water. The seedlings were grown in an incubator (HPS 1500, Heraeus Vötsch, Hanau, Germany) in the dark for 6 days at 25 °C and 80–85% relative humidity. Salt stress was imposed by substituting water with 0.21 M NaCl, which induces a severe stress on the basis of durum wheat salt tolerance [66], whereas, to estimate the osmotic component of salt stress, 0.42 M mannitol solution iso-osmotic with the salt solution was used. In the experiment reported in Figure 9, the growth solution was supplemented with 1 mM PG. Germination and growth parameters were determined on fresh material, whereas the biochemical assays and the RNA extraction were carried out on the shoots harvested and stored at −80 °C.

### 3.2. Seed Germination and Seedling Growth Measurements

The germination percentage was recorded daily up to 7 DAS, using the extrusion of a 0.2 cm long radicle as a criterion. The mean germination time (MGT) expressed in days was calculated by using the following equation:

MGT = Σ(D × n)/Σn(1)

where D is the number of days from the onset of germination, n is the number of seeds that had germinated on day D and Σn is the total number of germinated seeds [67]. Root and shoot growth in terms of lengths was measured and expressed as mean of 10 plants for each of the three replicates used for each treatment.

### 3.3. Measurement of the MDA Content

The MDA content was measured using a modified method based on Zhang and coworkers [68]. Briefly, durum wheat shoots of control and stressed seedlings from 2 to 6 DAS were homogenized under nitrogen, using a pre-chilled mortar and pestle. Three hundred mg of ground samples were resuspended in 5 mL 5% trichloroacetic acid and centrifuged at 12,000× *g* for 15 min at 4 °C. Two mL of the supernatant were mixed with 5 mL of 5% thiobarbituric acid and the mixture was incubated in boiling water for 15 min and then cooled on ice to stop the reaction. The absorbance of the supernatant was measured at 450, 532 and 600 nm and the MDA content was calculated and expressed as µmol *per* g dry weight (DW) of shoot tissue.

### 3.4. Measurement of the Superoxide Anion Generation Rate

The rate of the superoxide anion generation was determined according to Misra and Fridovich [69] by measuring the oxidation of epinephrine to adrenochrome. Briefly, durum wheat shoots of control and stressed seedlings from 2 to 6 DAS were homogenized under nitrogen, using a pre-chilled mortar and pestle. One gram of ground samples was resuspended in 10 mL cold 50 mM Na phosphate buffer (pH 7.0) and centrifuged (twice) at 12,000× *g* at 4 °C for 15 min. Total protein content of the crude shoot extract was determined by a modified-Lowry assay [70], using bovine serum albumin as the standard. Five hundred µg of crude shoot extract were added to a reaction mixture containing 1 mM epinephrine in 2 mL 50 mM Na phosphate buffer (pH 7.0) and the rate of the superoxide anion generation was measured by following the absorbance increase at 480 nm due to the epinephrine conversion to adrenochrome (ε_480 nm_ = 4.00 mM^−1^·cm^−1^). The rate was expressed as µmol superoxide anion produced *per* min *per* g DW of shoot tissue.

### 3.5. Expression Analysis of the TdLpx Genes

Extraction of the total RNA was carried out from the shoots of control and stressed seedlings from 2 to 6 DAS using Trizol^®^ reagent (Thermo Fisher Scientific, Waltham, MA, USA), and the first-strand cDNA was synthesised from 1 µg total RNA using the SuperScript™ II RNase H- reverse transcriptase (200 E.U., Thermo Fisher Scientific, Waltham, MA, USA) and random primers. The first-strand cDNA was used as the template to amplify the fragments corresponding to the *TdLpx* transcripts using the specific primer pairs reported in Table 2.

As regards the *TdLpx-B1* genes, the cv. ‘Ofanto’ possesses the haplotype III that bears the *TdLpx-B1.1* and *TdLpx-B1.2* genes [36]; so, for the expression analysis of these genes, the primer pairs were designed on the corresponding full-length transcript sequences (NCBI GenBank accession numbers HM126475 and HM126472, respectively) previously isolated by our research group [36]. For the expression analysis of the *TdLpx-2* and *TdLpx-3* genes, the primer pairs were designed on the coding portions of the partial gene sequences isolated by Carrera and coworkers [35] (NCBI GenBank accession numbers DQ448001-DQ448004).

The PCR was performed with Go-Taq DNA polymerase (Promega, Madison, WI, USA) as follows: 5 min initial denaturation at 94 °C, then 28 cycles of 1 min at 94 °C, 30 s at 62 °C to 72 °C, and 1 min at 72 °C, followed by 5 min final extension at 72 °C. The PCR normalization was carried out by amplification of the constitutive *actin1* gene using the primer pairs reported in Table 2. The PCR was performed with Go-Taq DNA polymerase (Promega) as follows: 5 min initial denaturation at 94 °C, then 28 cycles of 1 min at 94 °C, 30 s at 62 °C, and 1 min at 72 °C, followed by 5 min final extension at 72 °C. The PCR products were separated on agarose gel, stained with Gel Red (Biotium, Inc., Fremont, CA, USA) and analyzed under UV light. The amplified PCR products were cloned and sequenced on both strands to confirm their identity.

### 3.6. Isolation of the Full-Length TdLpx-A2 Transcript

To identify the full-length sequence of the *TdLpx-A2* gene, A BLAST search was carried out in the Interomics cv. ‘Svevo’ genome browser (http://d-gbrowse.interomics.eu/gb2/gbrowse/Svevo/) using as query the full-length sequence of the bread wheat *TaLOX2* gene (NCBI GenBank accession number GQ166691) [71]. Two *Td-Lpx-2* genes were identified on chromosome 5A and 5B, which shared more than 99% identity with the partial sequences isolated by Carrera and coworkers [35] and corresponding to the *TdLpx-A2* and the *TdLpx-B2* genes (NCBI GenBank accession numbers DQ448002 and DQ448001, respectively).

Extraction of the total RNA was carried out from the shoots of control seedlings at the 2nd DAS and first-strand cDNA was used as the template to amplify the full-length *TdLpx-A2* transcript (NCBI GenBank accession number MT843321) using the primer pair 5′-ATGTTCGGCGTCGG CGGCATCG-3′ (forward) and 5′-TCAGATGGAGATGCTGTTGGGGATGC-3′ (reverse) designed on the *TdLpx-A2* gene identified in the genome of the cv. ‘Svevo’. The PCR was carried out using the Taq High Fidelity Phusion (Thermo Fisher Scientific) as follows: 2 min initial denaturation at 94 °C, then 35 cycles of 10 s at 98 °C, 30 s at 66 °C, and 30 s at 72 °C, followed by 7 min final extension at 72 °C. The PCR product was separated on agarose gel, stained with Gel Red (Biotium, Inc.) and analyzed under UV light. The amplified PCR product was cloned and sequenced on both strands to confirm its identity.

### 3.7. Heterologous Expression of the TdLOX2 in E. coli

For the heterologous expression of the TdLOX2 enzyme in *E. coli*, the ORF of the *TdLpx-A2* transcript was cloned using the Gateway system (Thermo Fisher Scientific, Waltham, MA, USA). Briefly, the ORF of the *TdLpx-A2* gene was cloned into the pCR8/GW/TOPO entry vector using the TA cloning and then transferred into the pDEST17 destination vector through an LR recombination reaction. The resulting expression clone pDEST17-6 × His-TdLOX2 was used to transform TOP10 (DH5α) competent *E. coli* cells (Thermo Fisher Scientific) that were grown overnight on Luria-Bertani (LB) agar plates containing 100 µg mL^−1^ ampicillin. Positive clones were identified by PCR and sequenced on both strands to verify that the ORF was ligated in-frame with the 6 × His tag. A single colony was used to inoculate LB medium (10 mL) containing 100 µg mL^−1^ ampicillin and the culture was incubated at 37 °C with shaking until the optical density at 600 (OD_600_) nm reached 0.5. This pre-culture was used to inoculate 500 mL fresh LB medium containing 100 µg mL^−1^ ampicillin to obtain a new culture with an OD_600_ of 0.05–0.1 that was incubated at 37 °C with shaking, until it reached the mid-log phase (OD_600_ ≅ 0.4). Then, the expression of the recombinant protein was induced by the addition of 0.2% (*v*/*v*) L-(+)-arabinose and, after incubation at 25 °C for 15 h, 100-mL culture aliquots were centrifuged at 5000× *g* for 15 min at 4 °C. The cell pellets were frozen under liquid nitrogen and stored at −80 °C until purification of the recombinant protein.

Separation of the *E. coli* protein fractions was carried out using CelLytic B Plus protein extraction kit (Sigma-Aldrich, Merck, Darmstadt, Germany). Briefly, roughly 2 g of transformed *E. coli* BL21-AI (DE3) cells were resuspended in CelLytic B Plus working, the suspension was incubated at room temperature with shaking for 15 min, and then centrifuged at 1900× *g* for 15 min. The soluble fraction present in the supernatant was precipitated with solid ammonium sulphate (70% saturation) and the precipitate was collected by centrifugation at 5000× *g* for 15 min at 4 °C, and dissolved in 2 mL 50 mM Na phosphate buffer (pH 7.5) containing 500 mM NaCl and 30 mM imidazole (Buffer A). The suspension was dialyzed overnight at 4 °C to remove the residual ammonium sulphate, whereas the insoluble material was removed by centrifugation at 10,000× *g* for 10 min at 4 °C. The recombinant protein was purified by loading the resulting preparation onto a 4-mL Ni-nitrilotriacetic acid (Ni-NTA) affinity column pre-equilibrated with buffer A. After two washes with buffer A containing 50 mM imidazole, the recombinant protein was eluted with buffer A containing 300 mM imidazole. Five-hundred-microliters fractions were collected and their protein content was measured by using a modified-Lowry assay [70], with bovine serum albumin as the standard. Fractions containing the highest concentrations of protein were pooled and dialyzed against 50 mM Na phosphate buffer (pH 7.0) containing 150 mM NaCl and 20% glycerol.

### 3.8. Measurement of the LOX Activity

The LOX activity was assayed essentially as reported in Pastore and coworkers [38] by measuring the absorbance increase at 234 nm due to the conversion of polyunsaturated fatty acids into the corresponding hydroperoxide (ε_234_ = 28 mM^−1^·cm^−1^). Unless otherwise specified, the reaction mixture consisted of 2 mL 50 mM Na phosphate buffer (pH 7.5) containing 1 mM linoleate. The reaction was started by the addition of 0.02–0.05 mg crude shoot extract or 1–5 µg TdLOX2 recombinant protein. One E.U. corresponded to the formation of 1 µmol conjugated diene *per* min at 25 °C. GRAFIT 5.0 software (ERITHACUS, from Sigma-Aldrich, Merck, Darmstadt, Germany) was used to analyse the data.

### 3.9. Analysis of the TdLOX2 Reaction Products

The identity of the reaction products of the recombinant TdLOX2 was determined according to the method described by Zhang and coworkers [72] with minor modifications by using an Agilent 1200 Series HPLC system (Hewlett-Packard, Waldbronn, Germany) equipped with a diode array detector. The enzymatic reaction was carried out as described above (see paragraph 3.8.) and after 30 min was stopped by the addition of 1N HCl; n-hexane was then added to the reaction mixture and the reaction products were extracted by shaking. Separation of the reaction products was carried out by SP-HPLC on a SUPELCOSIL LC-SI column (3 µm, 15 cm × 4.6 mm, Supelco Analytical, Sigma-Aldrich, Merck, Darmstadt, Germany). The eluent was composed of *n*-hexane/2-propanol/acetic acid (100:2:0.1, by volume) at a flow rate of 0.7 mL min^−1^ and the effluent was monitored at 234 nm, which indicated the conjugated diene system. To identify the TdLOX2 reaction products, their retention times were compared with those of authentic 13- and 9-HPOD isomers (Cayman Chemical, Ann Arbor, MI, USA).

## 4. Conclusions

In summary, although durum wheat is an important agro-economical crop, little is known about genes involved in its defensive functions against environmental stresses. The present study provides evidence that the *TdLpx-A2* gene is up-regulated by osmotic and high salinity stress and that its encoded TdLOX2 isoform plays a positive role in durum wheat response to hyperosomotic stresses probably acting through the control of ROS accumulation and the prevention of oxidative damages that occur in plants under stress. However, the molecular mechanism underlying the TdLOX2-mediated pathway under stress still remains unclear. This study provides a foundation for future investigations on overexpressed and/or knockout lines of the *TdLpx-A2* gene, as well as on the identification of TdLOX2-derived products. Comparison between tolerant, semi-tolerant, and susceptible genotypes will be also useful to answer the questions related to the physiological significance of this LOX isoform in durum wheat response to hyperosmotic stress.

## Figures and Tables

**Table 1:** Kinetic parameters of the purified recombinant TdLOX2.

Substrate	Km (µM)	Vmax (E.U./mg of Protein)
Linoleic acid	73.69 ± 7.33	35.42 ± 3.16
Linolenic acid	300.10 ± 21.36	7.41 ± 0.95
Arachidonic acid	741.00 ± 58.35	2.45 ± 0.51

**Table 2:** Primer pairs used to amplify *TdLpx* and *actin1* transcript fragments, annealing temperatures, and PCR product sizes.

Gene Fragment	Forward Primer (5′→3′)	Reverse Primer (5′→3′)	Annealing Temperature (°C)	Product Size (bp)
*TdLpx-B1.1*	CCAAGATGATACTGGGCGGGC	CGCCGCCTTGCCGTGGTTGG	62	1154
*TdLpx-B1.2*	TACACGCCGGTGCCGAGCGGCAG	CGTGTCACGCTGCCCGAGGTAGAG	72	912
*TdLpx-A2*	GACCTGACCACGGCGAAGAGCACC	TGCGGGCTCGATGGGTCCTCCACC	70	425
*TdLpx-B2*	GACCTGACCACCGCGAAGAGCACG	TTGTACACCGGGTGGGTCACGCTC	68	206
*TdLpx-A3*	AATACAGCACGGCGCGAAGAGCAC	GTTCACCCACCGCTCGATCGCCC	70	514
*TdLpx-B3*	CCTGCCGCACCCCCAGGGGATG	GTTCACCCACCGCTCGATCGCCC	70	532
*Actin1*	CTTCGGACCCAAGAAAGAAAGCC	CACCGCCCGTATTTCTCTAGTAGCC	62	280
